# Analysis and Design of a Compact Leaky-Wave Antenna for Wide-Band Broadside Radiation

**DOI:** 10.1038/s41598-018-35480-7

**Published:** 2018-12-10

**Authors:** Davide Comite, Symon K. Podilchak, Paolo Baccarelli, Paolo Burghignoli, Alessandro Galli, Al P. Freundorfer, Yahia M. M. Antar

**Affiliations:** 1 Sapienza University, Department of Information Engineering, Electronics and Telecommunications, Rome, Italy; 2 Heriot-Watt University, Institute of Sensors, Signals, and Systems, Edinburgh, United Kingdom; 3 Roma Tre University, Department of Engineering, Rome, Italy; 4 The Royal Military College of Canada, Kingston, Canada

## Abstract

A low-cost compact planar leaky-wave antenna (LWA) is proposed offering directive broadside radiation over a significantly wide bandwidth. The design is based on an annular metallic strip grating (MSG) configuration, placed on top of a dual-layer grounded dielectric substrate. This defines a new two-layer parallel-plate open waveguide, whose operational principles are accurately investigated. To assist in our antenna design, a method-of-moments dispersion analysis has been developed to characterize the relevant TM and TE modes of the perturbed guiding structure. By proper selection of the MSG for a fabricated prototype and its supporting dielectric layers as well as the practical TM antenna feed embedded in the bottom ground plane, far-field pencil-beam patterns are observed at broadside and over a wide frequency range, i.e., from 21.9 GHz to 23.9 GHz, defining a radiating percentage bandwidth of more than 8.5%. This can be explained by a dominantly excited TM mode, with low dispersion, employed to generate a two-sided far-field beam pattern which combines to produce a single beam at broadside over frequency. Some applications of this planar antenna include radar and satellite communications at microwave and millimeter-wave frequencies as well as future 5G communication devices and wireless power transmission systems.

## Introduction

Planar printed antennas have been receiving remarkable interest in the last few decades thanks to their ease of realization, cost effectiveness, and integrability with active circuitry^1^. As is well-known, the most common type, the resonant microstrip patch antenna, has consolidated design procedures but typically provides broad, far-field patterns with low to moderate gain and narrow operational bandwidths. Phased arrays using such patch antennas have to be designed in order to obtain more directive as well as scannable patterns, although at the expense of a considerable increase in design complexity and cost. This is because bulky and expensive feeding networks and phase shifters are typically required.

Printed leaky-wave antennas (LWAs) offer an attractive alternative to phased arrays for the synthesis of directive beams with a variety of pattern shapes and steering capabilities^2,3^. In particular, pencil beams scannable in the elevation and azimuth planes can be obtained with linear arrays of one-dimensional (1-D) LWAs^4^, whereas either conical scanned beams or broadside pencil beams are possible with two-dimensional (2-D) LWAs^3^. In both cases, the guided-wave (GW) and the nonresonant nature of the radiation mechanism can provide a wide operational bandwidth. However, the main-beam angle typically scans with frequency, a feature which may or may not be desired and depends on the application.

As concerns 2-D LWAs, an interesting class of annular structures is the so-called ‘bull-eye’ antenna, first introduced in^5^ and carefully examined in^6,7^ considering operation in the microwave range, and in^8–10^ at millimeter waves. A prototype working in the terahertz range has also been proposed in^11^. In general, these single-layer structures are constituted by an arrangement of concentric microstrip rings driven by a suitably designed surface-wave launcher (SWL) positioned at the centre of the antenna^12^.

The cylindrical TM_0_ surface-wave (SW) field excited by the SWL travels radially, and, due to the perturbation of the radially periodic metallic grating, transforms into a cylindrical leaky wave (LW). The annular grating is usually printed on a single-layer grounded dielectric slab (GDS) and the antenna synthesis is essentially based on a dispersion analysis of the cylindrical LWs supported by the structure. Due to the lack of translational invariance, which prevents a direct modal characterization of the entire structure, such a dispersion analysis is performed on a linearized version of the 2-D radial annular structure, i.e., on the corresponding 1-D (periodic) linear metal strip grating (MSG). The MSG strips are normal to the propagation direction of the relevant leaky mode, whose radiation features can be described in terms of a fast spatial harmonic. A detailed discussion about the effectiveness of this approach can be found in^7,13,14^, based on both far-field and near-field arguments.

**Figure 1:**
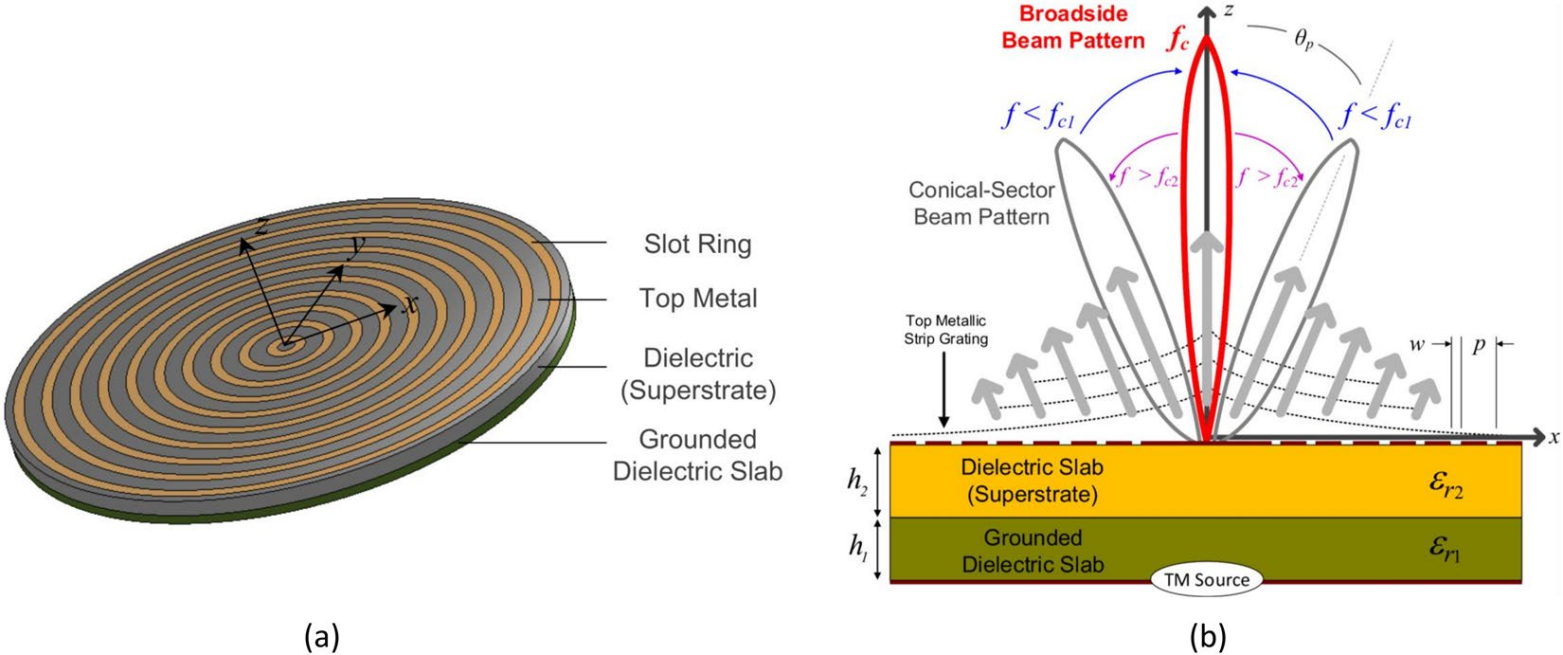
(**a**) Considered LWA defined by a MSG etched on top of a 2L-PPW; (**b**) Cross-sectional view of the proposed LWA. A finite slot in the ground plane can act as the planar bi-directional feed for TM cylindrical-wave excitation. A two-sided conical-sector beam pattern can also be observed below (*f* < *f*_c1_) and above (*f* > *f*_c2_) the broadside frequency range.

Following these developments, a *half-annular* version of a bull-eye antenna was recently reported by the authors in^15^, with the original structure briefly examined in^16^. Both designs were based on an annular microstrip grating placed on top of a dual- or two-layer (2L) grounded dielectric substrate (a 2L-GDS, as illustrated in Fig. 1(a,b)). A contrasting high-low profile for the dielectric constants was employed using commercial substrates. Due to the top metallic covering, the unperturbed (closed) guiding structure can be also described as a two-layer parallel-plate waveguide (2L-PPW). By this GDS and dielectric-superstrate configuration with top microstrip rings or slots for perturbation and radiation of the dominantly excited TM mode, and practically fed by a directive SWL integrated in the ground plane (as in^12,17^), a one-sided conical-sector and pencil-beam pattern was realized in^15^ with continuous frequency-scanning through broadside. Moreover, the antenna reported in^15^ can be described as a quasi-1-D LWA but with cylindrical-wave propagation within the low-profile guiding structure, due to the truncated and *half-annular* aperture of the antenna.

It is worth noting that the far-field radiation pattern and modal behavior for the 2-D planar periodic LWA in^15^ is similar to that of a one-sided 1-D periodic LWA with a mitigated open stopband. In fact, a single far-field pencil-beam pattern was achieved which continuously scanned, in the **E**(*x*−*z*) plane (see Fig. 1(b)), from the backward to the forward quadrant with an increase in frequency. This one-sided LWA allowed for continuous radiation through broadside with a minor reduction in the realized gain at broadside. This is mainly due to a double-symmetric bump of the normalized LW attenuation constant (*α*/*k*_0_) centered around the broadside radiating frequency^15^. Other works, focusing on 1-D periodic and quasi-uniform LWAs, have also studied such a desired scanning behavior (see e.g.^3,18–23^); whereas 2-D scanning LWAs based on metasurfaces have been proposed in^24–26^. Different LWA designs able to achieve broadside or frequency-scanning radiation with directive beam patterns in the far-field have been proposed in the last decade from microwave to optical frequencies (see e.g.^27–35^).

In this paper the 2L-PPW guiding structure from^15,16^ is employed to achieve persistent (i.e., wideband) and highly directional radiation at broadside whilst employing a bi-directional and integrated TM SWL feed system^7^. To this aim, the unperturbed and perturbed nature of the relevant bound and leaky modes of the 2-D ‘bull-eye’ responsible for radiation are fully reported and accurately analysed. Moreover, our proposed antenna design takes advantage of the preliminary discussions and supporting theory presented in^15,16^, and which are further developed here to describe the complete modal analysis and design of the proposed planar LWA offering wideband radiation. In this frame, a method-of-moments (MoM) formulation is also suitably adapted to describe the relevant radiating and guided TM and TE modes that can be supported by the structure. Relevant results for the background closed waveguide (i.e., the 2L-PPW and the 2L-GDS) are also discussed. All this makes the present work new and original with respect to^15^, and, with a unique design motivation. For example, in^15^ a different 2L-LWA was used to understand and optimize the dispersion of the relevant LW mode while also reporting the radiation performances. In particular, a directive pencil beam was observed in the far-field in^15^ which scanned through broadside as a function of frequency and where LWA feeding was realized by a uni-directional SWL.

The design methodology is based on the MoM in the spectral domain applied to an electric-field integral-equation (EFIE) formulation within the unit cell. In particular, we have employed a spectral-domain formulation of the MoM, in which the resulting matrix elements are expressed by integrals involving the planar components of the spectral dyadic Green’s function of the 2L-GDS. To this aim a suitable transverse network formalism is employed to describe the multi-layer structure (see, e.g.^36^, for all the relevant details on the approach). To our understanding, this has never been done before for this specific and low-cost dual-layer LWA configuration and this approach can also be applied to other types of multi-layer metal-strip-grating LWAs (consisting of two or more dielectric layers) as well as Fabry-Perot antennas.

**Figure 2:**
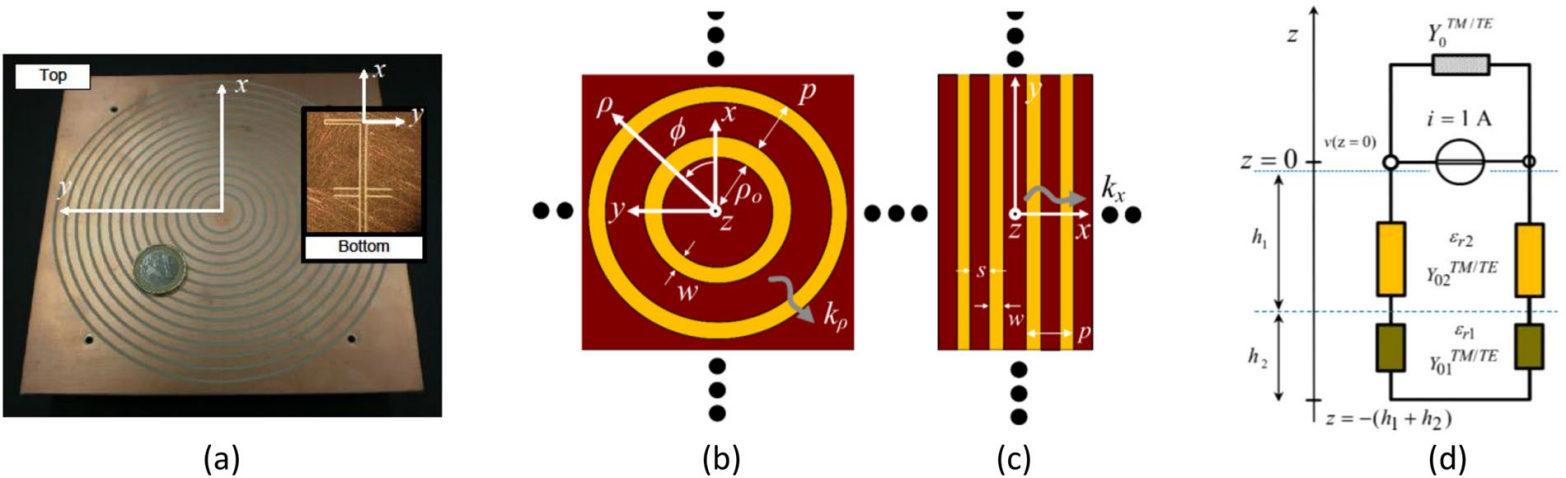
(**a**) Measured LWA prototype for K-band applications with a top MSG defined by 14 annular slots (*w* = 1.4 mm, *p* = 5 mm, *ρ*_0_ = 9.3 mm). The LWA is fed by a nondirective SWL placed at the origin, with a 50-Ω coplanar waveguide transmission-line feed system. The LWA (illustrated in Fig. 1) was realized using a high/low dielectric profile of commercially available substrates: *ε*_r1[2]_ = 10.2[3], *h*_1[2]_ = 1.27 mm (or 50 mil) [1.52 mm (or 60 mil)]. (**b**) The complex radial wavenumber, *k*_*ρ*_, of the 2-D LWA can be approximated by the LW propagation constant of an infinite structure with phase advancing normal to the MSG: (**b**) The infinite ‘bull-eye’ structure with radial propagation; (**c**) linear infinite analogue. (**d**) Transverse equivalent network for the evaluation of the spectral Green’s function of the background 2L-GDS. On the top a shunt admittance represents the open space, whereas on the bottom the horizontal line represents a short circuit on the ground plane at *z* = −(*h*_1_ + *h*_2_).

Numerical full-wave results using a commercial simulator and measurements of a prototype are also newly reported in this paper to assess the performance of the proposed LWA. Mainly to ensure that the employed TM mode for leaky-wave (LW) radiation has a zero cutoff frequency, is moderately dispersive, and operates within a unimodal regime over a significantly wide operating bandwidth. In addition, experiments confirm for the first time that enhanced broadside radiation characteristics are possible for such a simple and low-cost LWA. In particular, our fabricated prototype (see Fig. 2(a)) is capable of radiating a *fixed-angle* pencil beam at broadside over a significantly wide bandwidth of 8.7%, defining a new *two-sided* planar LWA. It should be made clear that, due to the combination of frequency-dependent conical-sector beam patterns, the physical operation of the antenna is still based on a two-sided frequency-scanned beam, with continued pencil-beam radiation at broadside.

For the first time numerical and experimental validations are reported on the role of the beam-splitting condition for such a compact (i.e., truncated) 2-D LWA. Thus we now bridge the connection with LW theory and the effects of a practically sized aperture. This further explains the achieved broadside radiating beam with a percentage bandwidth of more than 8.5%. To the best of the authors’ knowledge no similar 2L-LWA, with a rigorous analysis and the relevant supporting theory has been reported for this class of 2-D travelling-wave planar antenna structure which can offer simple and integrated feeding and efficient TM wave excitation for radiation.

## Methods

Scanning through broadside is typically problematic in more standard one-sided, 1-D periodic LWAs, due to the *LW open stopband* region^3,18,23^. However in^15^, broadside radiation was made possible by employing a unidirectional SWL positioned at the substrate periphery, which can be modeled as a horizontal magnetic dipole (HMD) antenna source in the ground plane. This HMD allows for broadside radiation provided that leakage from the antenna is optimized by removing or, at least, reducing the LW open stopband. To this aim, the top MSG aperture of the antenna in^15^, as well as its directive TM SWL and half-annular two-layer dielectric configuration, with the additional degree of freedom provided by the proper sizing of the dielectric superstrate layer, were suitably employed for antenna synthesis. On this basis a compact LWA offering a one-sided beam pattern scanning with frequency through broadside was obtained in^15^.

In more conventional periodic (or uniform) 2-D LWAs, the dominant cylindrical LW on the radial aperture generates a conical-sector beam pattern in the far-field where the main beam angle, ${\theta }_{p}\approx {\sin }^{-1}(\beta /{k}_{0})$ (being *β* the LW phase constant), scans with an increase in frequency towards broadside, as illustrated in Fig. 1(b). Directive radiation at broadside (*θ*_p_ = 0°) can be realized in the far-field within a frequency range (*f*_c1_, *f*_c2_) centered at *f*_c_. For such a periodic 2-D structure working on the *n* = −1 spatial harmonic, by increasing the frequency, the beam angle of the two-sided beam pattern first starts to reduce for *f* < *f*_c1_ until it coalesces into a single broadside pencil beam at the beam splitting frequency *f*_sp_ (given by *α* ≈ *β*_−1_)^34^, around *f*_c_. Typically radiation at broadside is obtained over a very narrow frequency range and can strongly deteriorate, mainly, due to the presence of a LW open stopband. This can introduce a considerable reduction of the realized gain for the LWA^7^. By further increasing the frequency above the open-stopband region for *f* > *f*_c2_, the beam splits again into a conical pattern (see Fig. 1(b)), gradually pointing far from broadside.

A similar narrow frequency range for broadside radiation is observed in uniform (or quasi-uniform) 2-D LWAs, where the beam angle of the two-sided beam pattern coalesces by decreasing the frequency until *f* = *f*_sp_ and then the gain quickly deteriorates by further decreasing the frequency below the cutoff of the leaky mode^34^. In contrast, the proposed 2-D LWA design under study overcomes these conventional limitations, being able to radiate a single pencil-beam consistently pointing at broadside (*θp* = 0°) over a wide radiating bandwidth, while also demonstrating more conventional frequency beam scanning off broadside. As discussed in the following sections, the main reason for this physical response is related to the mitigation of the open-stopband effects of the dominant TM leaky mode in the periodic 2L-PPW which has low dispersion, and to the existence of a less stringent beam-splitting condition for LWAs of finite length, as theoretically discussed in^35^.

### Theoretical Formulation

The reference planar periodic structure is a linear equi-spaced array of slots etched on the top surface of a 2L-GDS, i.e., a locally linearized version of the annular structure as illustrated in Fig. 2(c). The spatial period of the linearized structure is *p*, the width of each slot is *w* (or the width of each strip is *s* = *p* − *w*), the thicknesses and relative permittivities of the bottom substrate-dielectric and top superstrate-dielectric layers are *h*_1_, *ε*_r1_ and *h*_2_, *ε*_r2_, respectively.

Thanks to the 2-D nature of the problem, the spectrum of the propagating Bloch waves across the slots can be divided into both TM and TE modes. Each mode is characterized by a Floquet representation in terms of an infinite number of space harmonics with (generally complex) wavenumbers *k*_*xn*_ = *β*_*n*_ − *jα* = *β*_0_ + 2*πn*/*p* − *jα* (see the reference system in Fig. 2(c)), where, typically, the *n* = −1 spatial harmonic mainly contributes to radiation. In particular, the LW mode responsible for radiation from the proposed LWA is the dominant TM mode of the perturbed 2L-PPW, in a frequency range where the *n* = −1 space harmonic is fast, i.e., −*k*_0_ < *β*_−1_ < +*k*_0_. With ‘low’ attenuation rates (i.e., *α*/*k*_0_ < 0.1), directive beam patterns can be observed at beam angles defined by ${\theta }_{p}\approx {\sin }^{-1}(\sqrt{{\hat{\beta }}_{-1}^{2}-{\hat{\alpha }}^{2}})$, with |${\hat{\beta }}_{-1}$| ≥ $\hat{\alpha }$, where the hat $\hat{\cdot }$ indicates normalization with respect to *k*_0_.

### Design Guidelines

The 2L-GDS that constitutes the antenna substrate has to be properly designed to support the dominant TM mode for radiation. To this aim the permittivity of the substrates, their heights and the dimensions of the MSG should be properly sized. As concerns the substrate permittivity and thickness, their choice is mainly constrained by the SWL used to feed the proposed antenna. Such an antenna feeder, is fully planar and integrated into the bottom substrate and requires high values for the relative permittivity (${\varepsilon }_{{{\rm{r}}}_{1}}\approx 10$) and appropriate thickness (${h}_{1}\sqrt{{\varepsilon }_{{\rm{r}}}}/{\lambda }_{0}\approx 1/4$) for proper operation^7^. Moreover, the combined thickness and relative permittivities of the two dielectric layers has to be properly selected to generate an evanescent TM field in the top dielectric layer (or the dielectric superstrate region, defined by ${\varepsilon }_{{{\rm{r}}}_{2}}\le 3$ and ${h}_{2}\approx {\lambda }_{0}/2\sqrt{{\varepsilon }_{{{\rm{r}}}_{2}}}$). This ensures radial propagation within the bottom guide at the *h*_1_ and *h*_2_ interface of the 2L-PPW, similar to a TM_0_ SW mode that radially propagates at the air-dielectric interface of a GDS with an evanescent field component in the air region.

Once the two-layer structure is set, the dispersive behavior of the dominant TM mode can be determined while also analyzing its perturbed propagation due to an added MSG. This MSG can transform the TM mode into a fast LW that is responsible for directive radiation in the far-field. The slot width and the periodicity of the metal strips, which define the MSG, can be suitably tuned to obtain broadside radiation around a specific frequency and to provide sustained leakage, for example, such that *α*/*k*_0_ > 0.01 and where a double-symmetric bump around the open-stopband frequency, *f*_c_, is observed^15^. Furthermore, once the LW phase and attenuation constants are determined, one can further examine the Brillouin dispersion diagram of the guiding structure as well as its background waveguides, i.e., the relevant 2L-PPW and the 2L-GDS, whose dispersive features are investigated in the following sections. Following these developments the beam pointing angle in the far-field can be further characterized as well as the radiation performances of the developed LWA.

### Full-Wave Analysis of the Structure

To fully characterize the modal properties of the proposed structure, an efficient MoM code already developed by some of the authors^37,38^ has been modified to account for the presence of the two-substrate layers. This is achieved by exploiting the flexibility of the transverse network formalism. The approach is described as follows.

The periodicity allows for studying one single spatial period (*unit cell*). The modal surface density current **J**_*s*_ on the top single strip section within such unit cell can be represented as a linear combination of transverse and longitudinal components, *J*_*y*_(*x*) and *J*_*x*_(*x*), respectively. Hence we can write

1 $$\begin{array}{rcl}{{\bf{J}}}_{s}(\rho ) & \cong  & {{\bf{J}}}_{s}(x)={J}_{x}(x)\hat{{\bf{x}}}+{J}_{y}(x)\hat{{\bf{y}}}\\  & = & \sum _{q\mathrm{=0}}^{{N}_{x}-1}\,{A}_{q}{J}_{xq}(x)\hat{{\bf{x}}}+\sum _{r=0}^{{N}_{y}-1}\,{B}_{r}{J}_{yr}(x)\hat{{\bf{y}}}\end{array}$$

where $\hat{{\bf{x}}}$ and $\hat{{\bf{y}}}$ are the unit vectors of the Cartesian axes *x* and *y*, *N*_*x*_ and *N*_*y*_ are the number of basis functions used to represent the *x* and *y* components in the MoM formulation, and the complex coefficients *A*_q_ and *B*_r_ are the unknowns of the problem. The entire-domain basis functions adopted here, in particular, are^38^

2 $$\begin{array}{c}{J}_{x{\rm{q}}}(x)=j{U}_{{\rm{q}}}(\frac{2x}{s})\sqrt{1-{(\frac{2x}{s})}^{2}}\\ {J}_{y{\rm{r}}}(x)={T}_{{\rm{r}}}(\frac{2x}{s})\frac{1}{\sqrt{1-{(\frac{2x}{s})}^{2}}}\end{array}$$

where the functions *T* and *U* are Chebyshev polynomials of the first and second kind, respectively, and the square-root functions have been included in order to take into account the behavior of the current components near the edges at *x* = ±*s*/2 of the metal strip.

An integral equation can be obtained by enforcing that the tangential electric field vanishes on the strip within the unit cell. This can be completed by representing the electric field integral equation (EFIE) for the modal currents in the space domain, transforming the result into the spectral domain by using the Fourier transform, and then accommodating for an infinite number of *n* spatial harmonics. The integral equation and the corresponding electric-field expansion in the space domain, **E**(*x*, *z*), are as follows:

3 $$\hat{{\bf{z}}}\times \sum _{n=-\infty }^{+\infty }\,{\underline{\tilde{{\bf{G}}}}}^{ee}({k}_{xn})\cdot {\tilde{{\bf{J}}}}_{s}({k}_{xn})\,{e}^{-j{k}_{xn}x}=0\,\,{\rm{for}}\,\,|x| < w/2,$$

4 $${\bf{E}}(x,z)=\frac{1}{2\pi p}\sum _{n=-\infty }^{+\infty }\,{\underline{\tilde{{\bf{G}}}}}^{ee}(z,{k}_{xn})\cdot {\tilde{{\bf{J}}}}_{s}({k}_{xn}){e}^{-j{k}_{xn}x}$$

where ${\underline{\tilde{{\bf{G}}}}}^{ee}$ is the spectral dyadic Green’s function of the 2L-GDS for the electric field produced by an electric current source^36,39,40^ as illustrated in Fig. 2(c), and the tilde represents a Fourier transform with respect to *x*. The elements of the spectral Green’s function can customarily be determined in terms of the equivalent voltages and currents using the relevant transverse equivalent network. This is shown explicitly in Fig. 2(c) for our two-layer guiding structure under analysis.

By discretizing the integral equation within the unit cell (|*x*| < *p*/2), for both the transverse and longitudinal currents defined in Eq. (1), we get

5 $${\int }_{-s\mathrm{/2}}^{s\mathrm{/2}}[{J}_{xl}(x)\hat{{\bf{x}}}]\cdot \sum _{q=0}^{{N}_{x}-1}\,{A}_{q}\,\sum _{n=-\infty }^{+\infty }\,{\underline{\tilde{{\bf{G}}}}}^{ee}\,({k}_{xn})\cdot [{\tilde{J}}_{xq}({k}_{xn})\hat{{\bf{x}}}]\,{e}^{-j{k}_{xn}x}dx=0$$

for TM waves with *l* = 0, …, *N*_*x*_ − 1. Likewise for TE waves:

6 $${\int }_{-s\mathrm{/2}}^{s\mathrm{/2}}\,[{J}_{ym}(x)\hat{{\bf{y}}}]\cdot \sum _{r=0}^{{N}_{y}-1}\,{B}_{r}\,\sum _{n=-\infty }^{+\infty }\,{\underline{\tilde{{\bf{G}}}}}^{ee}\,({k}_{xn})\cdot [{\tilde{J}}_{yr}({k}_{xn})\hat{{\bf{y}}}]\,{e}^{-j{k}_{xn}x}dx=0$$

with *m* = 0, …, *N*_*y*_ − 1. Now Eqs (5) and (6) can be cast as a matrix linear system

7 $$[{Z}^{{\rm{TM}}}({k}_{x0})][A]=0\,{\rm{and}}\,[{Z}^{{\rm{TE}}}({k}_{x0})][B]=0,$$

by using the defined spectral currents ${\tilde{J}}_{x}$ and ${\tilde{J}}_{y}$ for both the TM and TE modes, respectively. The unknown complex wavenumber ${k}_{{x}_{0}}$ can be determined by calculation of the zero of the determinant for these matrices representing the eigenvalues of the linear system. The column matrices [*A*] and [*B*] contain the unknown coefficients for *A*_q_ and *B*_r_, respectively, whereas the MoM-matrix elements are defined as follows

8 $${Z}_{lq}^{{\rm{TM}}}({k}_{x0})=\sum _{n=-\infty }^{+\infty }\,{\tilde{J}}_{xl}(\,-\,{k}_{xn}){\tilde{G}}^{ee,xx}{\tilde{J}}_{xq}({k}_{xn})$$

9 $${Z}_{mr}^{{\rm{TE}}}({k}_{x0})=\sum _{n=-\infty }^{+\infty }\,{\tilde{J}}_{ym}(\,-\,{k}_{xn}){\tilde{G}}^{ee,yy}{\tilde{J}}_{yr}({k}_{xn})$$

for *l*, *q*[*m*, *r*] = 0, …, *N*_*x*_ − 1[*N*_*y*_ − 1]. The numerical evaluation of these slowly-converging spectral series can be effectively accelerated through the extraction and subsequent closed-form evaluation of their asymptotic values (for further details see^38^). Moreover, the unknown complex wavenumber for the fundamental mode *k*_*x*0_ = *β*_0_ − *jα* can finally be determined by locating the zeros of the determinant of the matrices Z^TM/TE^ in the complex plane, by suitably selecting the *proper* (ℑ{*k*_*zn*_} < 0) or *improper* (ℑ{*k*_*zn*_} > 0) nature of the relevant space harmonics. The vertical wavenumber in the air region *k*_*zn*_ is related to *k*_*xn*_ by the conventional separation condition.

### Transmission-Line Representation

As is known, the vertical propagation in the two-layer structure can be analyzed by reducing Maxwell’s equations to representative transmission-line equations^39^. Specifically, the electric and magnetic fields produced by the source can be expressed by means of voltages and currents on the transmission lines suitably excited by a unit amplitude, as shown in the dual-layer transverse equivalent network formulation (see Fig. 2(c)). By exploiting the spectral decomposition of the field for TM waves one can write^39^:

10 $$\begin{array}{ll}\frac{d}{dz}{V}^{TM} & =\,-\,j{k}_{z}{Z}^{TM}{I}^{TM}+{v}^{TM}\\ \frac{d}{dz}{I}^{TM} & =\,-\,j{k}_{z}{Y}^{TM}{V}^{TM}+{i}^{TM}\end{array}$$

where ${V}^{TM}={\tilde{E}}_{x}$, ${I}^{TM}={\tilde{H}}_{y}$, *Z*^*TM*^ = 1/*Y*^*TM*^ = *k*_*z*_/*ωε*, ${v}^{TM}=\,-\,{\tilde{M}}_{ye}$, ${i}^{TM}=\,-\,{\tilde{J}}_{xe}$, and ${\tilde{M}}_{ye}={\tilde{M}}_{y}-{k}_{t}/(\omega \varepsilon ){\tilde{J}}_{z}$, where *k*_*t*_ is the transverse wavenumber, i.e., normal to the *z* direction. The expression for the TE waves are omitted here for brevity. The relevant quantities can be described independently for each of the two dielectric layers and the air region for *z* > 0 as follows^36,40^

11 $$[\begin{array}{c}{\tilde{E}}_{x}\\ {\tilde{E}}_{y}\\ {\tilde{E}}_{z}\end{array}]=[\begin{array}{ccc}-\frac{{k}_{x}^{2}{\hat{V}}_{i}^{TM}+{k}_{y}^{2}{\hat{V}}_{i}^{TE}}{{k}_{t}^{2}} & \frac{{k}_{x}{k}_{y}({\hat{V}}_{i}^{TE}-{\hat{V}}_{i}^{TM})}{{k}_{t}^{2}} & \frac{{k}_{x}{\hat{V}}_{v}^{TM}}{\omega \varepsilon ({z}_{0})}\\ \frac{{k}_{x}{k}_{y}({\hat{V}}_{i}^{TE}-{\hat{V}}_{i}^{TM})}{{k}_{t}^{2}} & -\frac{{k}_{x}^{2}{\hat{V}}_{i}^{TE}+{k}_{y}^{2}{\hat{V}}_{i}^{TM}}{{k}_{t}^{2}} & \frac{{k}_{y}{\hat{V}}_{v}^{TM}}{\omega \varepsilon ({z}_{0})}\\ \frac{{k}_{x}{\hat{I}}_{i}^{TM}}{\omega \varepsilon (z)} & \frac{{k}_{y}{\hat{I}}_{i}^{TM}}{\omega \varepsilon (z)} & -\frac{1}{\omega \varepsilon (z)}[\frac{{k}_{t}^{2}{\hat{I}}_{v}^{TM}}{\omega \varepsilon ({z}_{0})}-j\delta (z-{z}_{0})]\end{array}]\,[\begin{array}{c}{\tilde{J}}_{x}\\ {\tilde{J}}_{y}\\ {\tilde{J}}_{z}\end{array}]\mathrm{.}$$

where *z*, *z*_0_ are the vertical abscissas of the field and source points, respectively.

On this basis, the evaluation of the spectral component of the relevant field quantity within each layer can be reduced to the calculation of voltages and currents produced on the equivalent transmission-line network. If only electric current densities are present (i.e., the currents on the metalizations), the electric field radiated by the structure is given by the matrix in Eq. (11). By means of the network formalism, the spectral dyadic Green’s functions ${\underline{\tilde{{\bf{G}}}}}_{ee}$, ${\underline{\tilde{{\bf{G}}}}}_{eh}$, ${\underline{\tilde{{\bf{G}}}}}_{he}$, ${\underline{\tilde{{\bf{G}}}}}_{hh}$ can be also determined^36^. We also note that an alternative approach would be to discretize the equivalent magnetic currents associated with the electric fields in the slots. In this case, an integral equation would be obtained by enforcing the continuity of the magnetic field across the slots and the resulting MoM matrix elements would involve the spectral Green’s dyadic ${\underline{\tilde{{\bf{G}}}}}_{hh}$. In any case, the only assumption for the two-layer structure relies on the homogeneous and isotropic nature of the considered dielectric materials.

For the 2L-GDS under analysis, excited by an electric line current of unit amplitude directed along $\hat{{\bf{y}}}$ (see the reference system in Fig. 1(b)) and placed on the metallic strip at *z* = *z*_0_ = 0, the impressed electric density current can be written as **J(r)** = $\hat{{\bf{y}}}$*δ*(*x*)*δ*(*z*). The nature of the source along the vertical *z*-direction allows one to associate a 1 A current generator as modeled in Fig. 2(d), where by solving the model through circuit theory one get $\hat{V}$(0) = 1/(*Y*_+_(*k*_*z*0_) + *Y*_−_(*k*_*z*1_, *k*_*z*2_)). *Y*_+_(*k*_*z*0_) and *Y*_−_(*k*_*z*1_,*k*_*z*2_) are the input admittances at the horizontal section *z* = 0 looking up and looking down, respectively, to be calculated for both the TE and TM modes. Here *Y*_−_ is a function of *k*_*z*1_ and *k*_*z*2_, which are related to the parameters of the two substrate layers defined by *h*_1_, *h*_2_, ${\varepsilon }_{{{\rm{r}}}_{1}}$, and ${\varepsilon }_{{{\rm{r}}}_{2}}$. A closed-form expression can be easily determined for ${Z}_{-}^{TM/TE}({k}_{z1},{k}_{z2})$ following standard transmission line theory. For the case at hand, i.e., for TM waves and recalling that ${\hat{V}}_{i}^{TM}=V\mathrm{(0)}$ in Eq. (11), the expression of the relevant Green’s function, ${\underline{\tilde{{\bf{G}}}}}_{ee}$, for the 2L-GDS can be obtained. A similar procedure can be applied to determine ${\underline{\tilde{{\bf{G}}}}}_{eh}$, ${\underline{\tilde{{\bf{G}}}}}_{he}$, ${\underline{\tilde{{\bf{G}}}}}_{hh}$, whose exact formulation is not required for the LWA under design in this paper.

## Discussion

**Figure 3:**
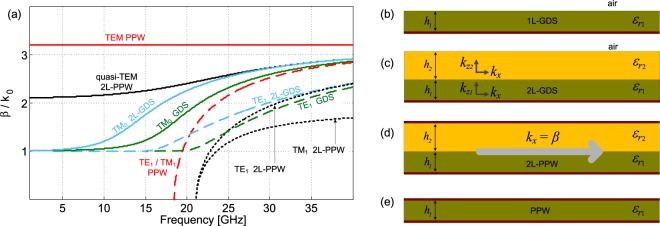
(**a**) TEM, TM and TE modal dispersion curves for the studied unperturbed structures (*ε*_r_1[2] = 10.2[3], *h*_1[2]_ = 1.27[1.52] mm). Green color, single-layer GDS (1L-GDS) (**b**) TM_0_ (solid line), TE_1_ (dashed line). Cyan color, 2L-GDS (**c**) TM_0_ (solid line), TE_1_ (dashed line). Black lines, 2L-PPW (**d**) quasi-TEM (solid line), TE_1_/TM_1_ (dotted line). Red lines, single-layer PPW (**e**) TEM (solid line), TE_1_/TM_1_ (dashed line).

To better understand the complex dispersion properties of the proposed 2L-PPW LWA, guided-wave (GW) propagation in different planar unperturbed (i.e., nonperiodic) structures were first studied as shown in Fig. 3, namely: (i) a single-layer grounded dielectric substrate (GDS or a 1L-GDS), (ii) a GDS with a dielectric superstrate having an air region above (a 2L-GDS), (iii) a parallel-plate waveguide (PPW) filled by two dielectrics (a 2L-PPW), and (iv) a PPW completely filled by a dielectric medium.

Possible modes are shown in Fig. 3(a) as well as the cross-sectional views of the unperturbed structures (see Fig. 3(b–e)). In the analysis, all values for the bottom layer were held constant (${\varepsilon }_{{{\rm{r}}}_{1}}=10.2$ and thickness *h*_1_ = 1.27 mm) while an air-dielectric interface, or a dielectric-dielectric interface and metal, was positioned on top when relevant (with thickness *h*_2_ = 1.524 mm and ${\varepsilon }_{{{\rm{r}}}_{2}}=3$). It should also be mentioned that both the 2L-GDS and the 2L-PPW can excite an evanescent field in the top superstrate layer, allowing for design control of the vertical attenuation constant of the TM guided wave, which can be described as a TM SW-like mode. This mode has been exploited for LW excitation and antenna radiation here and in^15,16^.

**Figure 4:**
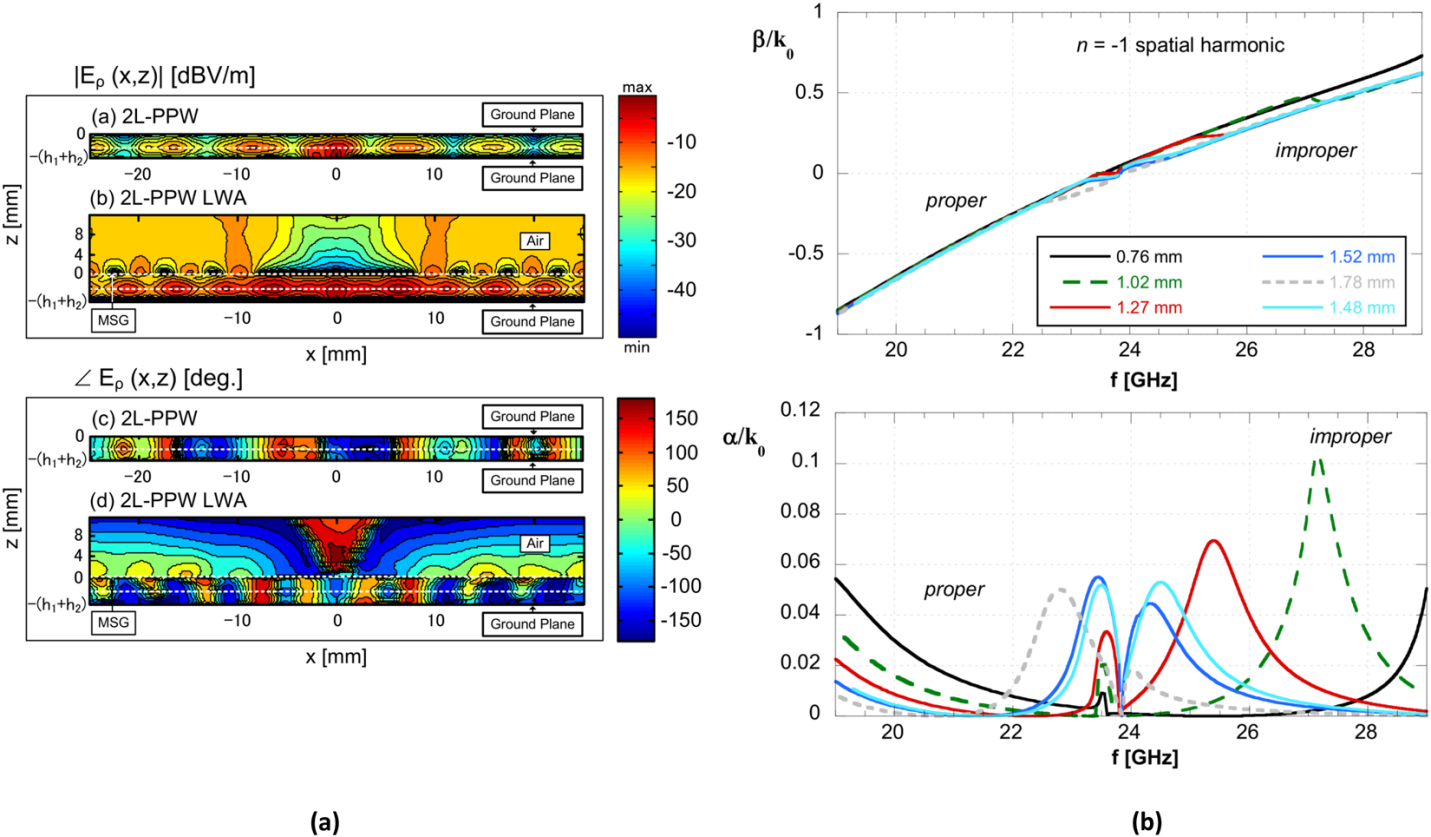
(**a**) Simulated amplitude (top panel) and phase (lower panel) for the electric field distributions at 23.3 GHz within the substrates and above the air/dielectric boundaries (interfaces defined by dotted white lines) for the nonradiating 2-L PPW (above) and the 2L-PPW LWA (below). Both simulated structures were excited by a nondirective SWL at the center and with a coplanar waveguide feeding line; (**b**) TM LW normalized phase and attenuation constants (upper and lower plot, respectively) defining the 2L-LWA (perturbed 2L-PPW) obtained using the MoM dispersion analysis on the linearized unit cell. Parameters: period *p* = 5 mm, slot width *w* = 1.4 mm, ${\varepsilon }_{{{\rm{r}}}_{1}\mathrm{[2]}}=\mathrm{10.2[3]}$, and *h*_1_ = 1.27 mm. A parametric analysis has been performed for different values of the superstrate thicknesses *h*_2_.

The TM SW-like mode can also be more formally defined as the quasi-TEM mode of the 2L-PPW (see Fig. 3): the simulated magnitude and phase of the electric field for this mode is shown in Fig. 4(a) (for comparison see also Fig. 6(b) in^16^, where the same distribution of the TM_0_ SW of a single layer GDS is reported). This (unperturbed) TM SW-like mode of the 2L-PPW is the fundamental mode of the supporting two-layer structure, has a zero cutoff frequency, and is moderately dispersive: its normalized phase-constant varies from about 2.1 to 2.9 over a 40 GHz bandwidth (see black curve in Fig. 3(a)). Conversely, all other comparative modes, i.e., the TM_0_ GDS and the TM_1_ PPW, vary from 1 and 0, respectively, to about 2.9 over the same frequency region.

We stress that the physical modal behavior of the 2L-PPW is considerably advantageous when designing the proposed 2L-LWA. In particular, by suitable sizing of the perturbing annular slots, with an increase in frequency, a slowly scanning beam can be realized. The simulated electric-field transverse distribution for this structure is shown in Fig. 4(a) (top and bottom panels indicating amplitude and phase, respectively) and compared with that of the LWA under analysis, whereas the dispersive behavior of the relevant *n* = −1 spatial harmonic is presented in the next section.

It is important to note that the TM SW-like modes, i.e., both the TM_0_ mode of the 2L-GDS and the quasi-TEM mode of the 2L-PPW are the *dominant* modes for these kind of structures (as shown in Fig. 3(a)). This is important when considering the operational frequency bandwidth for the practically designed nondirective TM_0_ SWL that was optimized to have more than a 13% impedance bandwidth (|*S*_11_| < −10 dB) centered at 23 GHz, when considering a single-layer GDS implementation^7^. This suggests that efficient coupling into both the 2L-GDS and the 2L-PPW is also possible for the considered bi-directional TM SWL (see Fig. 2(a) inset), mainly because the phase constants are of similar value and since the majority of the fields are contained at the dielectric-dielectric interface for the 2L-PPW. Overall, the modal behavior for this quasi-TEM mode of the 2L-PPW (see Fig. 3(a)), suggests that one can introduce a small unit -cell perturbation (i.e., *w* < *p*/2) within the top metallic sheet for TM LW excitation. Futhermore, this perturbation should also be large enough to generate appreciable values of the leakage rate for antenna radiation. A parametric analysis on the period *p* for the considered 2L-PPW will be presented in the next subsections.

The optimal design frequency for the considered two-layer antenna, dictated by the employed SWL, is fixed to 23 GHz. As discussed next, this frequency lies within a stopband region for the perturbed version of the TE_1_ mode of the employed 2L-PPW, which was a requirement for efficient TM_0_ SW excitation and to avoid spurious radiation. Therefore, this further suggests that similar dominant-mode coupling efficiencies are expected for the 2L-LWA, since the normalized phase constant behavior for the TE_1_ mode of the single-layer GDS is also very similar to the TE_1_ mode of the 2L-GDS at 23 GHz.

By selecting a proper design frequency, and employing commercially available dielectric substrates (see Fig. 2(a)) along with a practical SW feed system with a 50 -Ω connecting transmission line (i.e., the nondirective SWL), one ensures that the bottom dielectric layer can strongly support the selected and dominant TM mode of the guiding structure (i.e., the 2L-GDS or the 2L-PPW) for efficient LW excitation and radiation.

## Results

### LW Analysis of the Periodically-Loaded Guiding Structure

**Figure 5:**
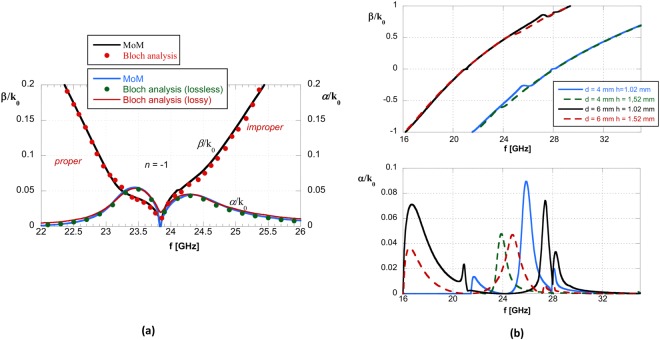
(**a**) TM LW normalized phase (absolute value) and attenuation constants for the practically realized and measured 2L-LWA (structure parameters: *p* = 5 mm, *w* = 1.4 mm, *ε*_r1[2]_ = 10.2[3], *h*_1[2]_ = 1.27[1.52] mm). Dispersion MoM results are also compared to Bloch analysis using CST full-wave simulations for the lossless case and while also considering substrate losses. In all cases a double-symmetric bump can be observed which is centered at the open LW stopband of about 23.8 GHz; (**b**) MoM dispersion analysis results for other periodicities along with other dielectric substrate thicknesses for the 2L-LWA. normalized phase and attenuation constant (upper and lower plot, respectively). Substrate parameters: ${\varepsilon }_{{{\rm{r}}}_{1}}=10.2$, *h*_1_ = 1.27 mm, and ${\varepsilon }_{{{\rm{r}}}_{2}}=3$. It can be observed that the double-symmetric bump is not clearly defined.

As is well-known, based on LW theory, the main properties of the antenna radiation pattern can be predicted through a careful inspection of the leaky-mode dispersion behavior of the periodically-perturbed guiding structure. As shown in Figs 4(b) and 5(b), the normalized phase constant of the considered *n* = −1 spatial harmonic increases *linearly* with frequency passing through zero, i.e., *β*_−1_/*k*_0_ = 0, at a frequency value *f*_sb_ around 24.7 GHz and defining a *proper* LW with a far-field pointing angle that will scan from backward endfire to broadside and an *improper* LW from broadside to forward endfire.

The LW dispersion analysis starts from the single-layer ‘bull-eye’ LWA design discussed in^7^ where a substrate having ${\varepsilon }_{{{\rm{r}}}_{1}}=10.2$ and thickness *h*_1_ = 1.27 mm were chosen, and then different superstrates having variable thickness were added on the top of the single-layer GDS. This starting point ensures that the impedance matching features of the aforementioned TM SWL^7^ are preserved for the 2L-LWA under study. Following this added superstrate variation, a parametric analysis is provided in Fig. 4(b) for a selection of two-layer structures capable of providing the required behavior for the LW attenuation constant around the stopband frequency *f*_sb_. As observed in Fig. 4(b), a fairly symmetric bump for *α*/*k*_0_ around *f*_sb_ is possible for the TM LW mode using a top substrate thickness of 1.48 mm and dielectric constant ${\varepsilon }_{{{\rm{r}}}_{2}}=3$. Fortunately, a dielectric thickness of 1.52 mm is commercially available and the TM LW mode for this structure can provide similar modal behavior.

Around the broadside frequency *f*_sb_ an open-stopband behavior is observed, where the attenuation constant *α* has a null point preceded or followed by a significant maximum. This behavior is typically responsible for a deterioration of the radiation performance for 1-D LWAs and the onset of undesired reactive effects. However, in most of the cases shown in Fig. 4(b) for our examined 2L-configuration, it can be observed that the maximum value of the normalized leakage constant is considerably lower than that obtained in the single-layer MSG (as commented in^15^, Fig. 3), and still allows for efficient radiation for the 2-D LWA. As shown in Fig. 4(b), for all other dielectric superstrate thicknesses, a symmetric bump around *f*_sb_ was not observed.

In Fig. 5(a) results obtained with the modal Bloch approach based on full-wave CST simulations of a finite number of unit cells are also provided. Good agreement is observed with the MoM dispersion analysis and CST (see, e.g.^15^, and references therein). Typically, the number of unit cells simulated depends on the complexity of the structure; for the case at hand, good results have been obtained with 15 cells. The agreement between the MoM and the hybrid Bloch-wave approaches is very good both for the proper and improper branches. Similar results are also shown for *α*/*k*_0_ when the substrate losses are included.

We note that the presence of a symmetric bump around *f*_c_, when also considering dielectric losses, permits to eliminate the null point of the attenuation constant. This allows for the mitigation of the open-stopband behavior, as also commented in^15^, and in addition determines a wide frequency band where |*β*_−1_| < *α* or |*β*_−1_| ≈ *α*. In particular, the possibility of almost equalizing the value of the attenuation constant (having values ranging from 0.025*k*_0_ to 0.05*k*_0_) around the phase constant null (i.e., around *f*_c_) for the *n* = −1 spatial harmonic, when also the open stopband is mitigated or possibly suppressed, can be suitable for obtaining continued broadside radiation in a *wide frequency band* for the two-sided 2-D LWA as proposed here. Dispersion curves for *β*_−1_/*k*_0_ and *α*/*k*_0_ for a superstrate having permittivity ${\varepsilon }_{{{\rm{r}}}_{2}}=3$ and different thickness *h*_2_ for the superstrate, as well as different periodicities for the MSG, are also shown in the parametric analysis of Fig. 5(b). Again, as in Fig. 4(b), it can be observed that the required double-symmetric bump for *α*/*k*_0_ is not obtained for any of these alternative configurations.

**Figure 6:**
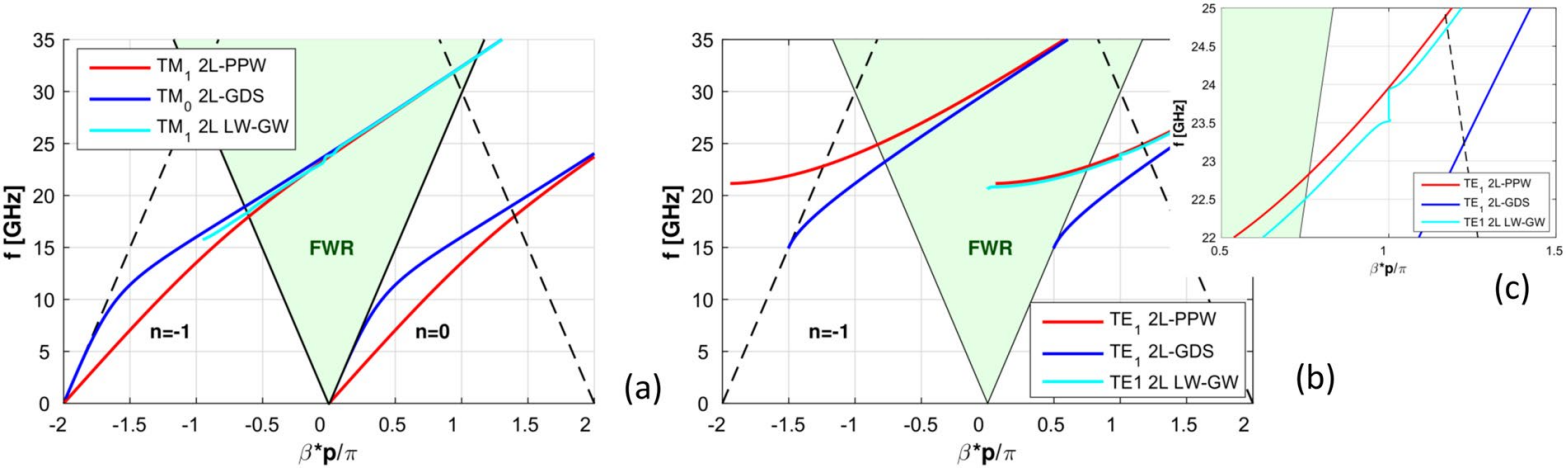
(**a**) Brillouin diagram for the 2D-LWA MSG considering TM waves (structure parameters as in Figs 3 and 5(a)). The blue curve corresponds to the fundamental (unperturbed) quasi-TEM mode of the 2L-PPW whereas the red curve corresponds to the TM_0_ mode for the 2L-GDS. The light blue curve is the *n* = −1 spatial harmonic for the TM mode (perturbed) of the 2L-LWA. In addition, the triangle regions are shown defining both the bound and radiating fast-wave region (FWR). (**b**) Brillouin diagram for the 2D-LWA MSG considering TE_1_ GWs both bound and radiating for the same structure parameters. The red [blue] 〈light blue〉 curve corresponds to the TE_1_ mode for the 2L-PPW (unperturbed) [2L-GDS (unperturbed)] 〈the 2L-LWA (perturbed)〉. (**c**) Confined range for the Brillouin diagram, i.e. defined frequency range for the designed and practically measured 2L-LWA. It can be observed that the perturbed TE_1_ mode is in a stopband regime and outside the FWR. This defines a reactive TE mode which is suppressed defining a suitable designed LWA for TM radiation and unimodal operation when considering broadside radiating frequencies.

The corresponding Brillouin diagrams for perturbed TM and TE modes are presented in Fig. 6(a,b). The periodicity for this MSG and the substrate values are representative of the fabricated 2L-LWA. In particular, the perturbed fundamental TM spatial harmonic (*n* = −1) and the phase constants of the two related spatial harmonics (unperturbed), supported by the insightful cases (i.e. the 2L-GDS and the 2L-PPW) for the 2L-LWA, are shown in Fig. 6(a), as was done in Fig. 3 for the dispersion curves of the unperturbed cases. The almost perfect linear scanning behavior inside the fast-wave region (FWR, depicted with a light green background) is clearly observable and confirms the effectiveness of the proposed design. In addition, the Brillouin diagram for the related TE cases is shown in Fig. 6(b). A confined range relevant to the TM broadside radiating frequency range for our proposed LWA (i.e., around 23 GHz) is also shown in Fig. 6(b). It can be observed that the perturbed TE_1_ mode (TE_1_ 2 L LW-GW) is in a stopband regime and outside the FWR when the dominant TM LW radiates^5^. This defines a reactive TE mode which does not contribute to antenna radiation.

It should be mentioned that similar antenna performances to that of our proposed LWA have been recently obtained for quasi-uniform 2-D LWAs, operating on the *n* = 0 spatial harmonic, in^41,42^. However, the symmetric behavior for the phase and attenuation constants was not observed. For our proposed LWA under study in this work, the radiating *n* = −1 spatial harmonic is in the proper and improper regions around *f*_c_, as shown in Fig. 5(a), which allows for a wider broadside radiation bandwidth. As a basis for comparison, a LWA based on a substrate integrated metamaterial presenting improved broadside radiation bandwidth has been also proposed in^43^. Even if this design shows a very good performance (1 dB radiation bandwidth of 4.2%), the underlying physical mechanism exploited to obtain broadside radiation is based on a 1-D design, which generates a fan-shaped beam being directive in just one specific plane. Similar performance has been obtained with the 1-D design proposed in^44^ using spoof plasmons to offer consistent gain, but with no ground plane, such that the LWA radiates a nearly omnidirectional beam with rotational symmetry around the longitudinal antenna axis. Also, a Fabry-Perot cavity antenna offering 6% broadside radiation bandwidth has been designed in^45^ and enhanced broadside radiation by means of a standing-wave LWA has also been recently presented in^46^.

Impressive results were also recently reported for an E-band corporate-fed slot array with a 17.2% broadside radiating bandwidth in^47^. That work was based on an involved and vertically stacked (multi-layer) corporate-feed slot array system which could be considered significantly involved to design, simulate, and optimize, as well as to numerically model. Moreover, the antenna fabrication and assembly process for this W-band slot antenna array and cavity-based structure might introduce some significant tolerance variations and thus cause some discrepancies between the simulated and measured performance. Regardless, the results in^47^ are impressive and suggest that with more layering and careful design of our proposed 2L-LWA, improved bandwidth may be possible.

Following these above discussions, we do feel that our proposed dual-layer bull-eye LWA represents a very good alternative with respect to the structures proposed in^43,44^. In fact, our design provides a pencil beam consistently pointing at broadside, in contrast with the fan beam or the omnidirectional beam provided by^43,44^, respectively. We would also like to stress that, since our 2-D LWA is based on a GDS with a fully integrated SWL feeding system, it can be considered more convenient when compared to^43,44^ for applications requiring integrated RF circuity and EM shielding effectiveness from the radiating aperture. This is because our SWL feed system is incorporated into the ground plane and on the backside of the antenna at its center and removed from any radiating element. This feed placement allows for simple RF circuit and IC ground plane integration for amplifiers, mixers, chip filters, etc. for communication applications, radar, and wireless power transmission systems.

### Antenna Simulations and Measurements

**Figure 7:**
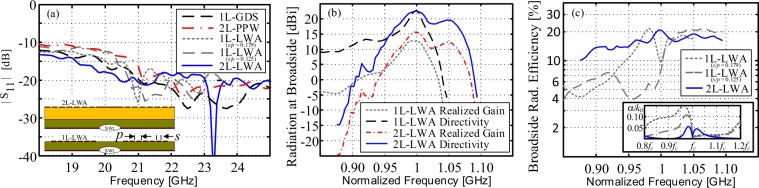
(**a**) Input reflection coefficient, in absolute value (dB), for various relevant structures and all fed by a non-directive SWL at the structure origin; i.e. a 1L-GDS and 2L-PPW (see inset and Fig. 3) as well as the examined 1L-LWAs from^7^ (with two different configurations of the MSG, as described in the figure inset) and the 2L-LWA of this work. (**b**) Directivity and realized gain at broadside, (defined for *θ* = *ϕ* = 0°) for the 1L-LWA s/p = 0.179 and the 2L-LWA as a function of frequency (and normalized to the frequency value for maximum realized gain at broadside for each LWA). (**c**) The radiation efficiency at the broadside versus the normalized frequency for the two 1L-LWAs from (**a**) and the 2L-LWA of this work. These results include dielectric and conductor losses as well as any mismatching losses for the compared LWAs. Also, this reported efficiency metric has been calculated by considering far-field values for when the radiated beam is at broadside only; i.e. 100% multiplied by the realized gain divided by the directivity for the dominant *E*_*θ*_ polarization whilst only considering values for *θ* = *ϕ* = 0° (broadside). It should be mentioned that the results for the more conventional total radiation efficiency, which considers radiation over all 3-D space and all possible losses, are well above 70% for both the 1L- and 2-LWA designs. The relevant LW attenuation constants directly computed by the MoM and considering no loss for structures, are also reported for comparison.

Figure 7(a) reports a comparison of the simulated input impedance matching of a 1L-GDS, a 2L-PPW, a 1L-LWA (with two different configurations of the MSG, as previously examined by the authors in^7^ and described in the figure inset), and the 2L-LWA of this work. All the structures are fed by the same non-directive SWL, which provided very good matching over a wide impedance bandwidth, and regardless of the top structure. To better appreciate the improved broadside radiation, Fig. 7(b) reports a comparison between the directivity and the realized gain of the 1L-LWA and the 2L-LWA versus the normalized frequency at broadside. As expected the 2L-LWA design of this work provides improved performance, in particular, an enhanced radiation bandwidth at broadside (i.e. *θ* = *ϕ* = 0°) when compared to the 1L-LWA. Finally, 7(c) reports the radiation efficiency, at broadside (again for *θ* = *ϕ* = 0°), versus the normalized frequency, for both the 1L-LWA (for two different configurations of the MSG) and the 2L-LWA. The latter shows more persistent (i.e., wide-band) broadside radiation, which can also be physically explained by comparing the LW attenuation constants (see the Fig. 7(c) inset in the bottom right corner) and observing the double-symmetric ‘bump’ provided by the 2L-LWA implementation. This configuration is able to provide sustained TM leakage and radiation over a wider frequency range when compared to the single-layer topologies.

**Figure 8:**
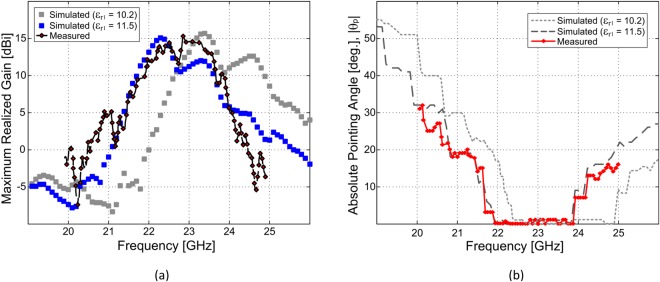
(**a**) Measured and simulated maximum realized gain in the **E**(*x*–*z*) plane (**a**) and the beam-pointing angle (**b**) for our fabricated 2L-LWA (see Fig. 2(a)). A 5-dB radiating bandwidth of about 8.7% can be observed in the measurements and simulations for the main beam positioned at broadside (when considering ${\varepsilon }_{{{\rm{r}}}_{1}}=11.5$). This also corresponds to the main beam being positioned at broadside (see Fig. 8(b)). It is interesting to note that the observed gain variations around the broadside radiating frequencies (between 23 GHz and 25 GHz for the full-wave simulations, for example, with ${\varepsilon }_{{{\rm{r}}}_{1}}=10.2$) are related to the double-symmetric bump of the LW attention constant. This is consistent with the numerical results in Fig. 5(a).

The 2L-LWA prototype presented in Fig. 2(a) and simulated in Fig. 7 was also measured in a calibrated anechoic chamber. The measured and simulated maximum realized gain and the beam pointing angle versus frequency are shown and discussed. As clearly visible in Fig. 8(a,b), a frequency shift can be observed between the simulated and measured curves when considering a dielectric constant of the bottom GDS equal to ${\varepsilon }_{{{\rm{r}}}_{1}}=10.2$, whereas a very good agreement is obtained when ${\varepsilon }_{{{\rm{r}}}_{{\rm{2}}}}=11.5$ is used in the full-wave simulation. This is due to the tolerance and anisotropy for the relative dielectric constant for the commercial substrate^48–50^ and is consistent with the results for the single-layer bull-eye LWA previously reported by some of the authors (for example, see Fig. 17 from^7^), since the exact same substrate was employed again for our new 2L-LWA (i.e., by removing the radial microstrip top rings by wet chemical etching). Specifically, the same bottom dielectric slab and ground plane, and thus the same TM SWL from^7^, were explicitly employed for the bottom layer of the antenna under study in this paper. Then, the top dielectric-superstrate and MSG were affixed to this original GDS.

Regardless of these features, measurements and full-wave simulations generally show a consistent gain and pointing angle profile versus frequency in Fig. 8(a,b). Also, in the open stopband frequency range, a minor reduction in the realized gain is observed at broadside in both the measurements as well as the simulations (see Fig. 8(a)) demonstrating consistent results. However, it should be noted that the experimental results in Fig. 8(a,b) show a minor discrepancy with the full-wave simulations for frequencies around 23 GHz. This could be related to some practical variations in the relative dielectric constant for the top dielectric layer as well as some minor fabrication and assembly tolerance errors for the measured prototype. Thus some minor discrepancies between the measurements and the full-wave simulations, due to these practicalities, can generally be expected when operating at microwave and millimeter-wave frequencies.

**Figure 9:**
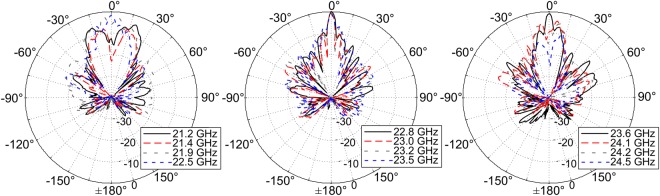
Measured beam patterns for the 2L-LWA in the ***E***(*x*–*z*) plane (referenced to the main slot of the non-directive SWL at the origin), where a two-sided beam pattern can be observed as a function of frequency as well as a directive beam at broadside. Results are normalized to the observed maximum at 22.8 GHz. For additional comparisons of the simulated and measured beam patterns, see Fig. 2 in^16^.

**Figure 10:**
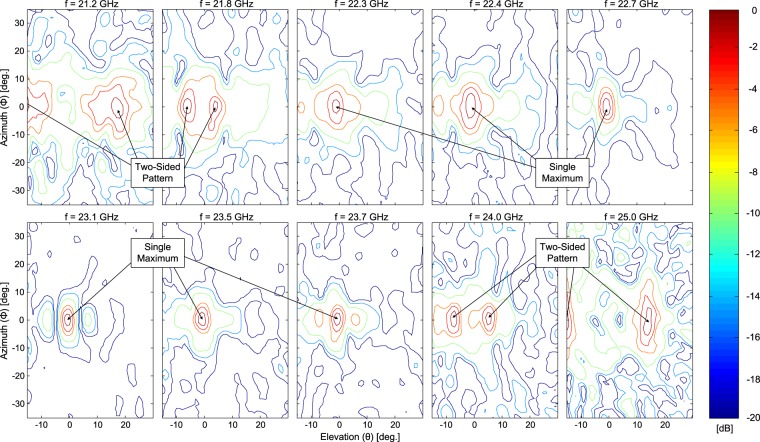
Measured 2-D far-field patterns in the azimuth and elevation. A single pencil-beam pattern at broadside is observed over an 8.7% bandwidth with conical-sector frequency beam scanning below and above 21.9 GHz and 23.9 GHz. Values shown in dB units and normalized to the observed maximum at each frequency.

Measured beam patterns normalized to the observed maximum at 22.8 GHz as well as 2-D contour gain patterns in the azimuth and elevation planes are reported in Figs 9 and 10, respectively. It is possible to appreciate the single pencil-beam pattern observed at broadside from about 22 GHz to about 23.7 GHz (see Fig. 10), confirming the noted bandwidth of about 8.7% as described in Fig. 8(a). For this broadside frequency range, and over the operating bandwidth of the antenna, sidelobe levels are generally less than 10 dB below the main beam maximum (but in a worst case about 7 dB) which may be acceptable for certain communication applications. Additional measurements and simulations for the fabricated 2L-LWA can be found in^16^ where measured 1-D and 2-D realized gain plots were provided for other frequencies along with additional comparisons to full-wave simulations.

We note in Fig. 8(a,b) that the obtained radiation bandwidth at broadside extends over almost 2 GHz. This result exceeds what is expected on the basis of the modal dispersion analysis only (see Fig. 5(a), where *β* ≤ *α* from 23.2 GHz to around 24 GHz with ${\varepsilon }_{{{\rm{r}}}_{1}}=10.2$ and from 22.3 GHz to around 22.8 GHz with ${\varepsilon }_{{{\rm{r}}}_{1}}=11.5$, whose relevant dispersion curve is essentially a down-shifted version of the same curve and it is not shown here for brevity). Interestingly, in^35^ it has been observed that for LWAs of *finite length* the beam-splitting condition is not strictly given by the condition *β* ≈ *α*, valid in the case of a LWA with infinite length, but by *β* ≈ *n*_*s*_*α*, with *n*_*s*_ ≥ 1 and *n*_*s*_ reaching values of 6 or more for pratical LWAs.

**Figure 11:**
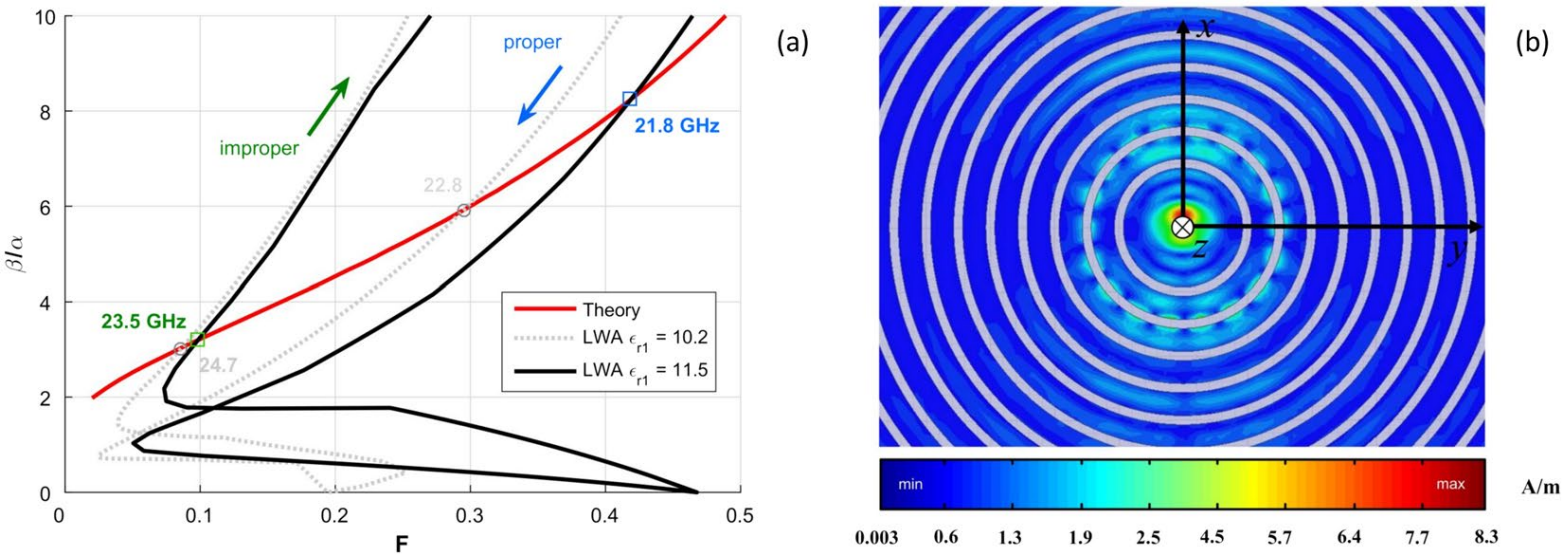
(**a**) Plot of *β*/*α* vs. *F* as in^35^ for the proposed bull-eye antenna having ${\varepsilon }_{{{\rm{r}}}_{1}}=10.2$ (dotted gray curve) and ${\varepsilon }_{{{\rm{r}}}_{1}}=11.5$ (solid black curve). The blue and green arrows show the direction on the curve corresponding to increasing values of the frequency, going from the proper to the improper branch of the involved fast spatial harmonic. The colored numbers show the corresponding frequencies at the intersection points; (**b**) Simulated current distribution on the top radial aperture of the 2L-PPW LWA. Azimuthally directed currents can be observed at 20.5 GHz.

In Fig. 11(a), the theoretical curve showing the behavior of *β*/*α* versus *F*, the ratio of the power remaining at the ends of the LWA and the input power (indicated here with *F* as in^35^) is reported in red: the intersection points (blue on the proper branch and green on the improper one, 21.8 GHz and 23.5 GHz, respectively) of this curve with that relevant to the proposed design allow us to predict the frequency range for which broadside radiation is generated by the finite-length (i.e., truncated) LWA. The theoretical range (i.e., 22.8 GHz to 24.7 GHz) obtained with the ‘ideal’ value for the relative dielectric constant (${\varepsilon }_{{{\rm{r}}}_{1}}=10.2$) revealed by the dotted gray curve in Fig. 11(a) is in very good agreement with the simulated result shown in Fig. 8(b) (see the corresponding dotted gray curve). However, the theoretical frequency range (i.e., 21.8 GHz to 23.5 GHz) obtained with the ‘actual’ (${\varepsilon }_{{{\rm{r}}}_{1}}=11.5$) permittivity revealed by the solid black curve in Fig. 11(a) are in good agreement with both the simulated and measured results in Fig. 8(b) for the absolute value of the beam pointing angle (dashed dark gray and solid red curves).

By comparing the results presented in Figs 10 and 11(a), it is possible to observe a small frequency shift between the experimental and theoretical limit range of frequencies for broadside radiation. This is mostly likely due to the dielectric constant in the vertical direction of the practical substrates, which can be different than in the horizontal direction^48–50^. This anisotropy, which can be significant for thick substrates, is a result of manufacturing and shows a frequency dependence (see^48^, p. 758, Appendix A for an quite exhaustive discussion on these aspects). As discussed in^48^ by increasing the value for the dielectric constant (a similar procedure is reported in^7^) to achieve better agreement between the simulations and the measurements (see Fig. 8(a,b)). Regardless of these studies, the relevant broadband behavior is very well predicted by the extended beam-splitting condition for the considered and truncated LWA. We further stress that the condition *β* ≈ *α* is still valid for LWA design since it predicts the peak of the maximum realized gain^35^, as is confirmed by the maximum value of the gray curve in Fig. 8(a), obtained at around 23.3 GHz. This is in agreement with the condition *β* ≈ *α* observed in Fig. 5(a).

It is interesting to note that a ‘staircase-like’ function for the beam pointing angle is observed in both the measurements and simulations for Fig. 8(b), similar to^7^. However, the nonlinear scanning behavior is observed for off-broadside frequencies only. For example, from about 20.2 GHz to 20.5 GHz the beam pointing angle in Fig. 8(b) is fixed at ±40° (considering ${\varepsilon }_{{{\rm{r}}}_{1}}=10.2$); moreover, the normalized LW attenuation constant is small, i.e., *α*/*k*_0_ < 0.01, which implies that the LW field may not be the dominant field on the antenna aperture. The mentioned ‘staircase-like’ function in the beam pointing angle can in fact be related to the presence of azimuthal current distributions generated by the slot ring modes, as depicted in Fig. 11(b) where such surface currents are plotted at 20.5 GHz on the top metallic aperture of 2L-LWA.

A very similar response was observed in^7^ for the constituent microstrip rings. We stress that these resonances for the 2L-LWA under study are related to the presence of the coplanar waveguide feeding line connected to the nondirective SWL. This is because a TEM mode is generated on the feedline (from the substrate periphery) with power guided to the planar TM source positioned at the origin. More specifically, the *E*_*z*_ field lines of the transmission line can be aligned with that of the relevant field component for the radial slot ring modes. However, their contributions to the radiated far-field are negligible (see also^7^). This can be observed in the measurements and simulations at 20.5 GHz as the realized gain is below 2 dBi for all cases in Fig. 8(a), and, less than −5 dBi for the simulations when ${\varepsilon }_{{{\rm{r}}}_{1}}=10.2$.

## Conclusion

A dual-layer radial metal slot-grating planar antenna providing two-sided conical-sector and pencil beam patterns with a wide bandwidth for broadside radiation has been proposed. By means of a full-wave dispersion analysis for the reference structure, the complex modal behavior has been described. Through this modeling, optimized parameters for the 2L-PPW and MSG have been selected in order to mitigate the open stopband effects of the leaky mode responsible for radiation. The capabilities of the finite-length LWA, in providing persistent and continued broadside radiation over a wide frequency range, have been experimentally assessed and related to the more relaxed beam splitting conditions which characterize truncated LWA structures of practical size. Measured maximum gain values greater than 15 dBi are observed at broadside. The final design results in a compact, low-cost, and low-profile 2L-LWA prototype demonstrating consistent broadside radiation over more than an 8.5% wide-bandwidth.
